# Discovery and characterization of the tubercidin biosynthetic pathway from *Streptomyces tubercidicus* NBRC 13090

**DOI:** 10.1186/s12934-018-0978-8

**Published:** 2018-08-28

**Authors:** Yan Liu, Rong Gong, Xiaoqin Liu, Peichao Zhang, Qi Zhang, You-Sheng Cai, Zixin Deng, Margit Winkler, Jianguo Wu, Wenqing Chen

**Affiliations:** 1 State Key Laboratory of Virology, and College of Life Sciences, Wuhan University, Wuhan, 430072 China; 2 Key Laboratory of Combinatorial Biosynthesis and Drug Discovery, Ministry of Education, and School of Pharmaceutical Sciences, Wuhan University, Wuhan, 430071 China; 3 Austrian Centre of Industrial Biotechnology, Petersgasse 14, 8010 Graz, Austria

**Keywords:** Biosynthesis, Tubercidin, 7-deazapurine, Phosphoribosylpyrophosphate, NADPH-dependent reductase, Nudix hydrolase, Synthetic biology

## Abstract

**Background:**

Tubercidin (TBN), an adenosine analog with potent antimycobacteria and antitumor bioactivities, highlights an intriguing structure, in which a 7-deazapurine core is linked to the ribose moiety by an *N*-glycosidic bond. However, the molecular logic underlying the biosynthesis of this antibiotic has remained poorly understood.

**Results:**

Here, we report the discovery and characterization of the TBN biosynthetic pathway from *Streptomyces tubercidicus* NBRC 13090 via reconstitution of its production in a heterologous host. We demonstrated that TubE specifically utilizes phosphoribosylpyrophosphate and 7-carboxy-7-deazaguanine for the precise construction of the deazapurine nucleoside scaffold. Moreover, we provided biochemical evidence that TubD functions as an NADPH-dependent reductase, catalyzing irreversible reductive deamination. Finally, we verified that TubG acts as a Nudix hydrolase, preferring Co^2+^ for the maintenance of maximal activity, and is responsible for the tailoring hydrolysis step leading to TBN.

**Conclusions:**

These findings lay a foundation for the rational generation of TBN analogs through synthetic biology strategy, and also open the way for the target-directed search of TBN-related antibiotics.

**Electronic supplementary material:**

The online version of this article (10.1186/s12934-018-0978-8) contains supplementary material, which is available to authorized users.

## Background

**Fig. 1:**
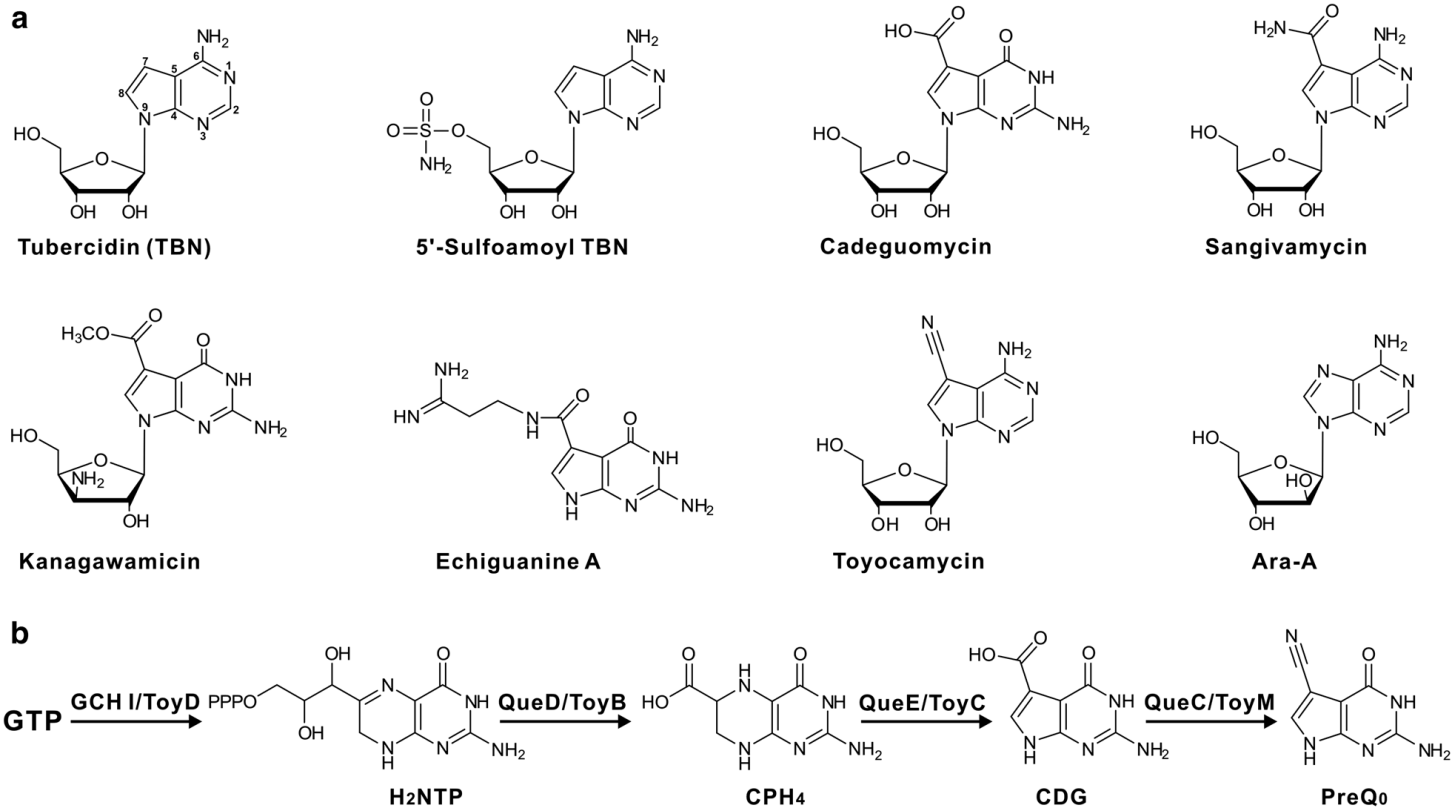
Structures of TBN and related antibiotics and the confirmed biosynthetic pathway to PreQ_0_. **a** Chemical structures of TBN and related purine nucleoside antibiotics. TBN is produced by *S. tubercidicus* NBRC 13090; 5′-sulfamoyl TBN is produced by *S. mirabilis;* cadeguomycin is produced by *S. hygroscopicus*; sangivamycin/toyocamycin is produced by *S. rimosus* ATCC 14673; kanagawamicin is produced by *Actinoplanes kanagawaensis*; echiguanine A is produced by *Streptomyces* sp. MI698-50F1; Ara-A (arabinofuranosyladenine) is produced by *S. antibioticus* NRRL 3238. **b** The confirmed biosynthetic pathway leading to PreQ_0_. A four-enzyme cascade for the synthesis of PreQ_0_ is identified in both queuosine and sangivamycin/toyocamycin biosynthetic pathways. GCH I, GTP cyclohydrolase I (ToyD); QueD/ToyB, 6-carboxy-5,6,7,8-tetrahydropterin (CPH_4_) synthase; H_2_NTP, 7, 8-dihydroneopterin-3′-triphosphate; QueE/ToyC, 7-carboxy-7-deazaguanine (CDG) synthase; QueC/ToyM, 7-cyano-7-deazaguanine (PreQ_0_) synthetase

Pyrrolopyrimidine (also known as 7-deazapurine) containing compounds have been discovered to be widely distributed in nature as microbial secondary metabolites (SMs) and also as hypermodified base (queuosine) in RNA [1, 2]. Usually, this family of SMs shows diverse biological functions, ranging from cofactors (involved in the biosynthesis of tetracycline antibiotics) as well as DNA repair to antibiotics with herbicidal, antibacterial, antiviral, antifungal, antitumor, and antineoplastic activities [1, 2]. Previously, toyocamycin/sangivamycin (produced by *Streptomyces rimosus* ATCC 14673) and tubercidin (TBN, produced by *Streptomyces tubercidicus* NBRC 13090) served as prototypes for the deazapurines (Fig. 1a) [2–4]. Since their discovery, a series of structurally-related antibiotics has been continuously isolated from terrestrial and marine sources with guidance of bioactivity assays (Fig. 1a) [2].

Tubercidin is susceptible to phosphorylation by adenosine kinase to mono-, di-, and tri-phosphorylated forms accounting for its structural resemblance to adenosine [5, 6]. Accordingly, it is capable of acting as a powerful inhibitor of RNA and DNA polymerase, and shows a relatively-broad spectrum of bioactivities [2]. TBN is bioactive against *Candida albicans*, *Mycobacterium tuberculosis*, and *Streptococcus faecalis*, however, it exhibits no inhibition of other Gram positive bacteria and fungi [2, 5]. In addition, TBN shows antiviral and antitumor activities [5], and more interestingly, it can also kill trypanosomes by targeting glycolysis, especially by inhibition of phosphoglycerate kinase [7].

TBN features an unusual deazapurine core, in which N-7 is substituted for C-7 (Fig. 1a) [4]. Previous metabolic labeling experiments indicated that the deazapurine core is derived from a purine precursor, likely GTP, and C-1′, 2′, and 3′ of ribose are utilized to construct the pyrrole ring with elimination of C-8 in GTP [8, 9]. Subsequently, the enzymatic logic for the construction of deazapurine core has been deciphered by independent studies, which were focused on the identification of the biosynthetic pathway of queuosine and toyocamycin/sangivamycin (Fig. 1b) [2, 10]. A four-enzyme cascade has been demonstrated to be responsible for the biosynthetic steps leading to PreQ_0_ (7-cyano-7-deazaguanine) (Fig. 1b). Among them, GTP cyclohydrolase I (GCH I, ToyD) catalyzes the first step from GTP to H_2_NTP (7, 8-dihydroneopterin-3′-triphosphate) with opening the ribose ring, followed by a series of Amadori rearrangement and subsequent recyclization. The intermediate H_2_NTP is then sequentially recognized by CPH_4_ (6-carboxy-5, 6, 7, 8-tetrahydropterin) synthase (QueD/ToyB) and CDG synthase (QueE/ToyC) [2, 10]. The enzymatic mechanism of QueE/ToyC, a member of the radical *S*-adenosyl-l-methionine (SAM) protein, has been elucidated by elegant studies of the Bandarian group. This enzyme catalyzes a key SAM- and Mg^2+^-dependent radical-mediated ring contraction step [11, 12], which is common to the biosynthetic pathways of all deazapurine-containing compounds. More recently, QueC/ToyM has been verified as an amide synthetase, as well as a nitrile synthetase, which could accept both the acid and amide forms of CDG enabling sequential amidation and dehydration to the nitrile [13, 14].

Although the distinguished activities and unusual structure of TBN are well known, nature’s strategy for the building of this molecule has as yet remained poorly understood. In the present study, we have identified the TBN biosynthetic gene cluster from *S. tubercidicus* NBRC 13090 (*S. tubercidicus* hereafter) by engineered production of TBN in a heterologous host, and have further elucidated that TBN biosynthesis involves a PRPP-dependent assembly logic associated with tailoring reduction and phosphohydrolysis steps. Our deciphering of the TBN biosynthetic pathway provides a solid basis for the further combinatorial biosynthesis of this group of nucleoside antibiotics towards improved features, and opens the way for the target-directed genome mining of novel TBN-related antibiotics from the available microbial genome reservoirs.

## Results

### Identification of the TBN biosynthetic gene cluster

**Fig. 2:**
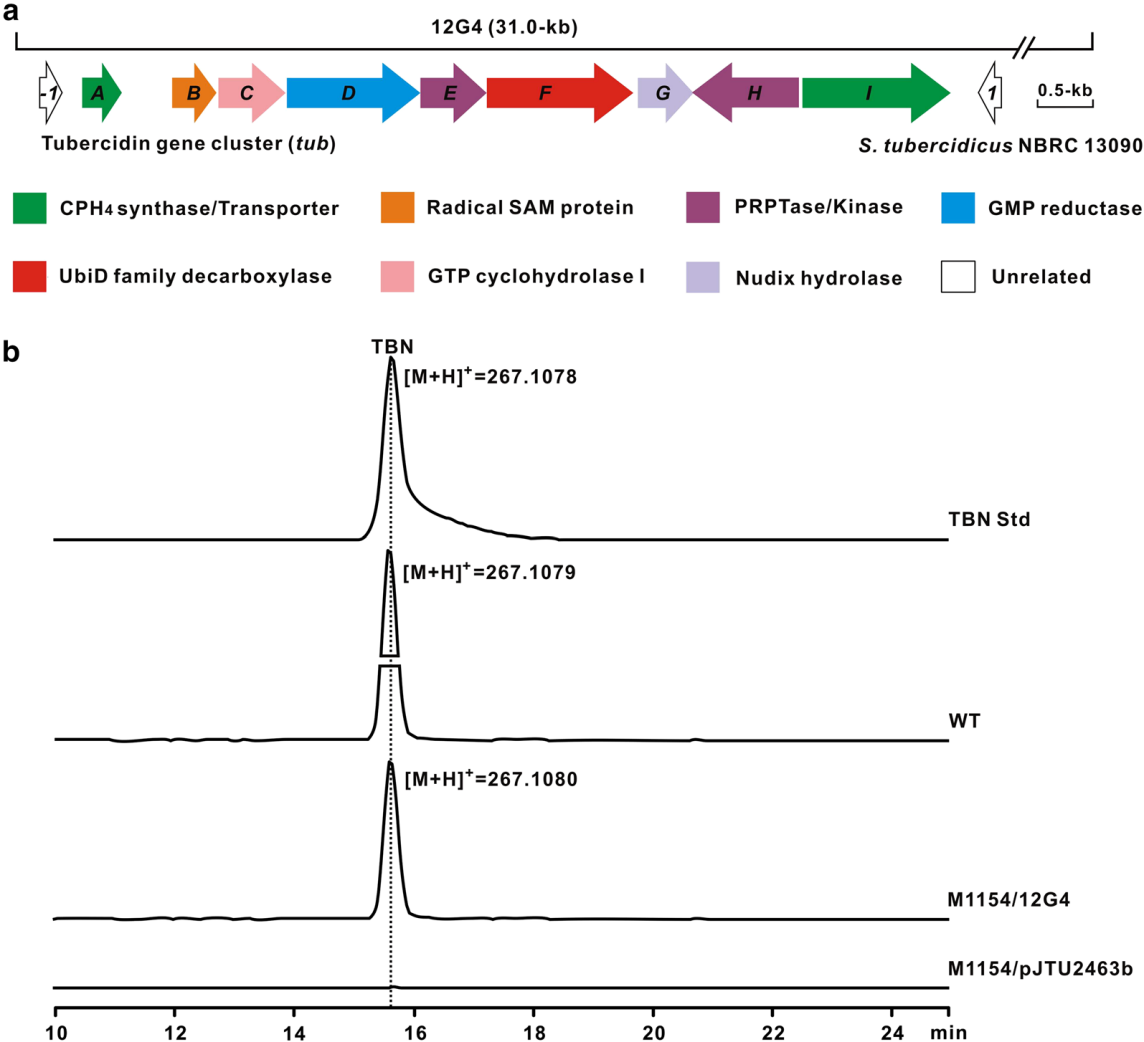
Genetic organization and validation of the gene cluster (*tub*) for TBN biosynthesis. **a** Genetic organization of the *tub* gene cluster. **b** LC–MS analysis of the target metabolite produced by the recombinants of *S. coelicolor* M1154. TBN Std, the authentic standard of TBN; WT, the metabolites of the wild type TBN producer *S. tubercidicus*; M1154/12G4, the metabolites of *S. coelicolor* M1154 derivative harboring the cosmid 12G4; M1154/pJTU2463b, the metabolites of *S. coelicolor* M1154 derivative harboring the plasmid pJTU2463b as negative control

To identify the gene cluster for TBN biosynthesis, the genome of *S. tubercidicus* was sequenced by an Illumina Hiseq method, rendering appr. 7.88-Mb of non-redundant bases after assembly of clean reads (Additional file 1: Table S1). The genomic data was then annotated by Glimmer 3.02 software, affording 7263 open reading frames (ORFs) (Additional file 1: Table S1). TBN features the deazapurine core, which is also existed in toyocamycin, sangivamycin, and other related antibiotics; we therefore deduced that the initial enzymatic steps for the building of the core of them follow an identical logic. We accordingly utilized ToyD (GTP cyclohydrolase I) and ToyB (CPH_4_ synthase) as the enzyme probes to conduct BlastP analysis against the genome of *S. tubercidicus*. As expected, a candidate gene cluster (*tub*) encoding enzymes involving TubC (64% identity to ToyD) and TubA (65% identity to ToyB) was identified from the genome (Fig. 2a, Table 1). Further looking through the surrounding region resulted in the identification of the genes coding for a radical SAM enzyme (TubB) and a GMP reductase (TubD) (Fig. 2a, Table 1). These results suggest that the *tub* gene cluster is very likely responsible for the biosynthesis of TBN.

**Table 1:** Deduced functions of the open reading frames in the *tub* gene cluster

Protein	aa	Proposed function	Homolog, Origin	Identity, similarity (%)	Accession no.
Orf-1	58	(2Fe-2S)-binding protein	AWI43_15300, *Streptomyces* sp. WAC04657	78, 83	KYG55615.1
TubA	125	CPH_4_ synthase	ToyB, *Streptomyces rimosus* ATCC 14673	65, 79	ACF06634.1
TubB	139	Radical SAM family protein	SPAR_33961, *Streptomyces sparsogenes* DSM 40356	71, 79	OMI34937.1
TubC	199	GTP cyclohydrolase I (GCH I)	ToyD, *Streptomyces rimosus* ATCC 14673	64, 75	ACF06635.1
TubD	385	GMP reductase	ToyE, *Streptomyces rimosus* ATCC 14673	61, 75	ACF06636.1
TubE	194	Phosphoribosylpyrophosphate transferase	ToyH, *Streptomyces rimosus* ATCC 14673	39, 53	ACF06639.1
TubF	422	UbiD family decarboxylase	ASE41_09730, *Streptomyces* sp. Root264	90, 93	KRD23282.1
TubG	168	Nudix hydrolase	ASE41_09725, *Streptomyces* sp. Root264	96, 96	KRD23281.1
TubH	314	Carbohydrate kinase family protein	ASE41_09720, *Streptomyces* sp. Root264	82, 86	KRD23280.1
TubI	374	MFS transporter	ASE41_09715, *Streptomyces* sp. Root264	83, 88	KRD23279.1
Orf1	68	Hypothetical protein	ASE41_09630, *Streptomyces* sp. Root264	41, 60	KRD23264.1

### Engineered production of TBN in the heterologous host *S. coelicolor* M1154

To correlate the *tub* gene cluster to TBN production, a cosmid 12G4 (with appr. 30.0-kb insertion DNA, Additional file 1: Table S2) harboring the TBN gene cluster was screened from the pJTU2463b-derived genomic library of *S. tubercidicus* (Additional file 1: Table S2), and then it was introduced into the heterologous host *S. coelicolor* M1154 [15] via conjugation. The validated conjugants were then fermented for further metabolite analysis, and LC–MS analysis indicated that the sample (M1154/12G4) is capable of producing the distinctive [M+H]^+^ ion of TBN at *m/z* 267.1080 (Fig. 2a). In addition, MS/MS analysis showed that the main fragments were generated at 134.8841, 248.9537, 231.1378, and so forth, fully consistent with those of the TBN authentic standard (Additional file 1: Figure S1). These combined data unambiguously demonstrate that the cosmid 12G4 confers the heterologous host *S. coelicolor* M1154 with the capability of TBN production.

### In silico analysis of the TBN biosynthetic gene cluster

In silico analysis showed that the TBN gene cluster spans appr. 8.0-kb continuous chromosomal region, and consists of 9 genes (*tubA*-*I*) (Fig. 2a, Table 1). Genes *tubCBA* are proposed to be responsible for the initial biosynthetic steps to CDG. The gene *tubC* codes for GTP cyclohydrolase I which shows 64% identity to ToyD of toyocamycin biosynthesis, and the product of *tubB* (radical SAM enzyme) exhibits 71% identity to SPAR_33961 of *S. sparsogenes* DSM 40356. *tubA* encodes CPH_4_ synthase that indicates significant similarity (65% identity) to ToyB in toyocamycin biosynthetic pathway. *tubD* encodes a GMP reductase (61% identity to ToyE) that is proposed to be responsible for IMP (Inosine-5′-monophosphate) biosynthesis, whereas TubE has 39% identity to ToyH, an enzyme hypothetically responsible for the assembly step during toyocamycin biosynthesis. TubF exhibits high similarity (90% identity to ASE41_09730 from *Streptomyces* sp. Root264) to UbiD family decaboxylases, which bear a recently identified cofactor prFMN (prenylated-FMN) [16–19], but the roles of most UbiD family decaboxylases are functionally unassigned. TubG is annotated as a Nudix hydrolase, and this family of enzymes usually plays “house-cleaning” role to sanitize nucleotide pool in primary metabolism [20]. *tubHI* individually code for carbohydrate kinase family protein (82% identity to ASE41_09720) and MFS (Major facilitator superfamily) transporter.

### TubE functions as a CDG-PRPP phosphoribosyltransferase

**Fig. 3:**
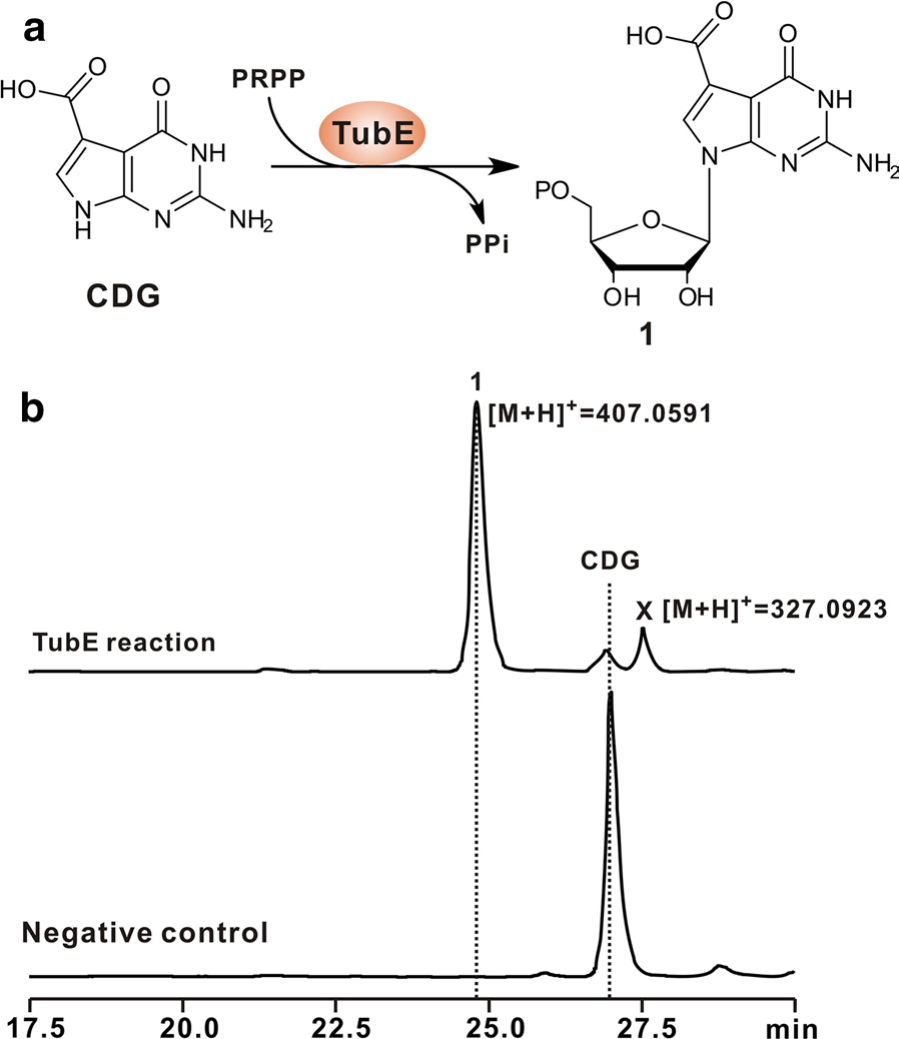
Biochemical characterization of TubE as a CDG-PRPP transferase. **a** Schematic of the TubE-catalyzed reaction in the presence of PRPP and CDG. **b** HPLC analysis of the TubE reaction. TubE reaction, HPLC profile of the TubE reaction using PRPP and CDG as substrates; Negative control, HPLC profile of the reaction using PRPP and CDG as substrates but without TubE added. Compound **X** shows [M+H]^+^ ion at *m/z* 327.0923, with main fragment ions at 177.9827, 194.9009, 292.0454, and 309.9970, which are fully consistent with the theoretical fragmentation pattern of dephosphorylated **1**

BlastP analysis indicates that *tubE* encodes a purine phosphoribosylpyrophosphate transferase (Additional file 1: Figure S2), which likely governs the assembly step during TBN biosynthesis. To determine if TubE fulfills such enzymatic role, we overexpressed and purified the protein to near homogeneity from *E. coli* (Additional file 1: Figure S3A), and tested its activity in vitro first, using deazaguanine and PRPP as substrates, and LC–MS results indicated that the TubE reaction could not generate the expected product (Additional file 1: Figure S3B, C), suggesting that this enzyme does not recognize deazaguanine as a substrate. As a result, we ponder whether the assembly step is prior to decarboxylation during TBN biosynthesis (Fig. 1b), namely, CDG is the prime substrate of TubE. We then re-examined TubE activity using this compound as substrate, and HPLC analysis showed that the TubE reaction is capable of generating a new peak at RT = 24.7 min, which is absent in the negative control (Fig. 3b). Further LC–MS analysis indicated that the target peak (RT = 24.7 min) could produce a characteristic [M+H]^+^ ion at *m/z* 407.0591 (Fig. 3b) with main fragment ions at 177.9070, 194.9660, 294.0352, and 389.0653, well matched to the theoretical fragmentation pattern of compound **1** (Additional file 1: Figure S3D). To finally clarify the chemical identity of the product (**1**) formed by TubE, it was purified and analyzed by nuclear magnetic resonance (NMR). The results showed that ^1^H, ^13^C, and 2D NMR signals of the compound are highly consistent with those of **1**, confirming that the TubE-catalyzed product is **1** (Additional file 1: Figures S4, S5). Taken together, all of the data demonstrate that TubE functions as a CDG-specific phosphoribosyltransferase.

### Biochemical characterization of TubD as an NADPH-dependent reductase

**Fig. 4:**
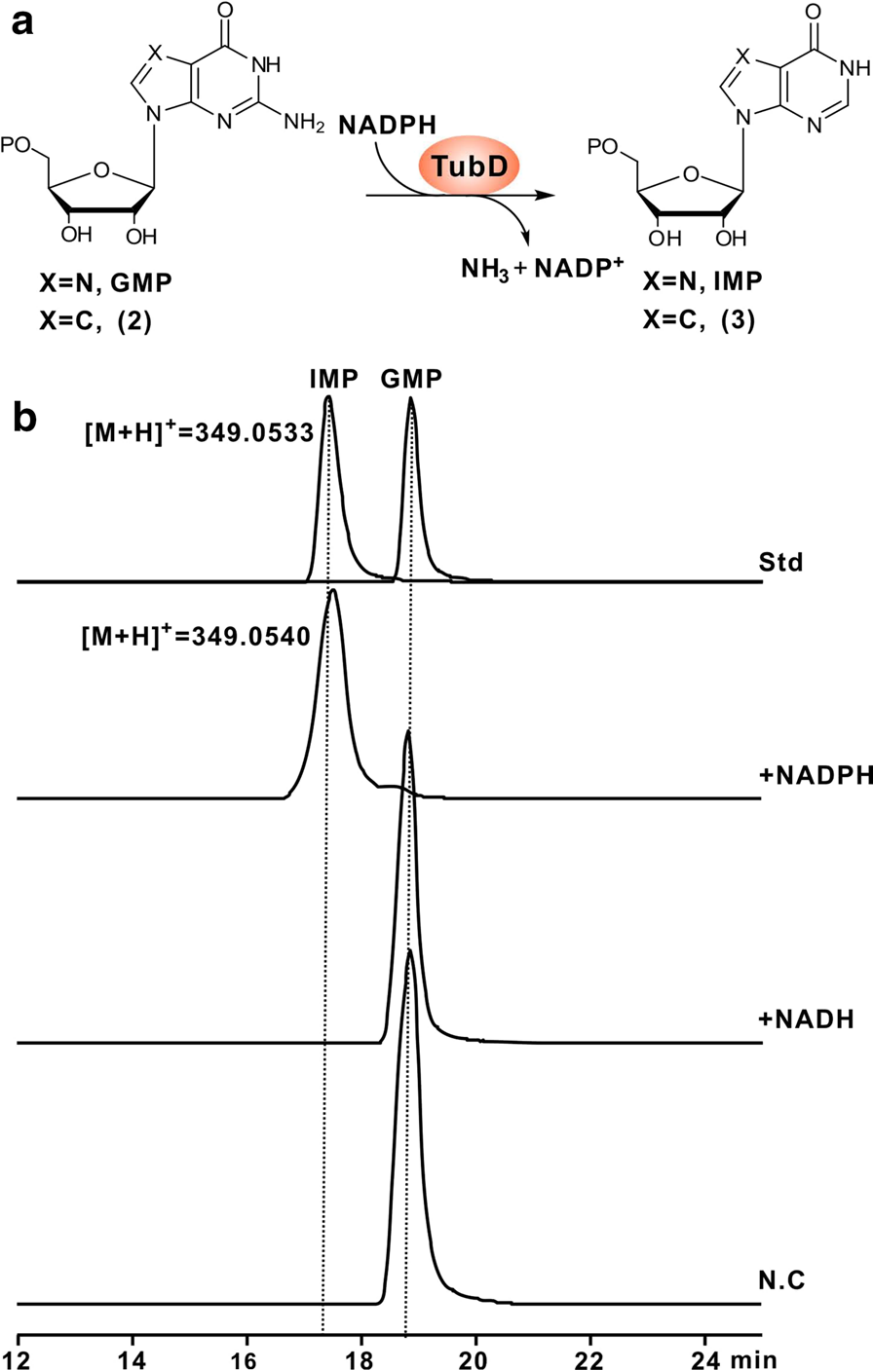
In vitro characterization of TubD as an NADPH-dependent reductase. **a** Schematic of the TubD-catalyzed reaction in the presence of the redox cofactor NADPH. **b** LC–MC analysis of the TubD reaction. Std, EIC analysis of the authentic standards of IMP and GMP; +NADPH, EIC analysis of the TubD reaction with NADPH as redox cofactor; +NADH, EIC analysis of the TubD reaction with NADH as redox cofactor; N.C, EIC analysis of the reaction with NADPH and substrate added but lacking the enzyme TubD

In silico analysis exhibited that TubD bears an IMP dehydrogenase/GMP reductase domain (Additional file 1: Figure S6), implicating that TubD should play a similar role in TBN biosynthesis (presumably converting compound **2** to **3**) (Fig. 4a). To see if TubD is capable of performing this function, it was expressed and purified from *E. coli* (Additional file 1: Figure S7A). We then tested its activity in vitro using GMP, an analog of **2**, as a substrate (Fig. 4a). LC–MS analysis showed the target IMP [M+H]^+^ ion at *m/z* 349.0540 in the TubD reaction, while it was absent in the negative control (Fig. 4b). In addition, MS/MS analysis indicated that the predominant fragment ions were generated at 118.8544, 136.8826, 233.0000, and 331.0666, fully consistent with those of the IMP authentic standard (Additional file 1: Figure S7B, C). Subsequently, we tested cofactor specificity for TubD activity, and LC–MS analysis showed that TubD reaction with NADH as cofactor could not give rise to the target IMP [M+H]^+^ ion, suggesting that this cofactor could not replace NADPH to supply reducing equivalents for the TubD reaction (Additional file 1: Figure S7D). Moreover, we also tested the factors that potentially affect TubD activity, and found that the metal ion K^+^ plays a pivotal role for TubD activity (Additional file 1: Figure S7D). These combined data suggest that TubD is an NADPH-dependent reductase catalyzing reductive deamination of compound **2** to **3** in TBN biosynthesis.

### Biochemical characterization of TubG as a tailoring Nudix hydrolase

**Fig. 5:**
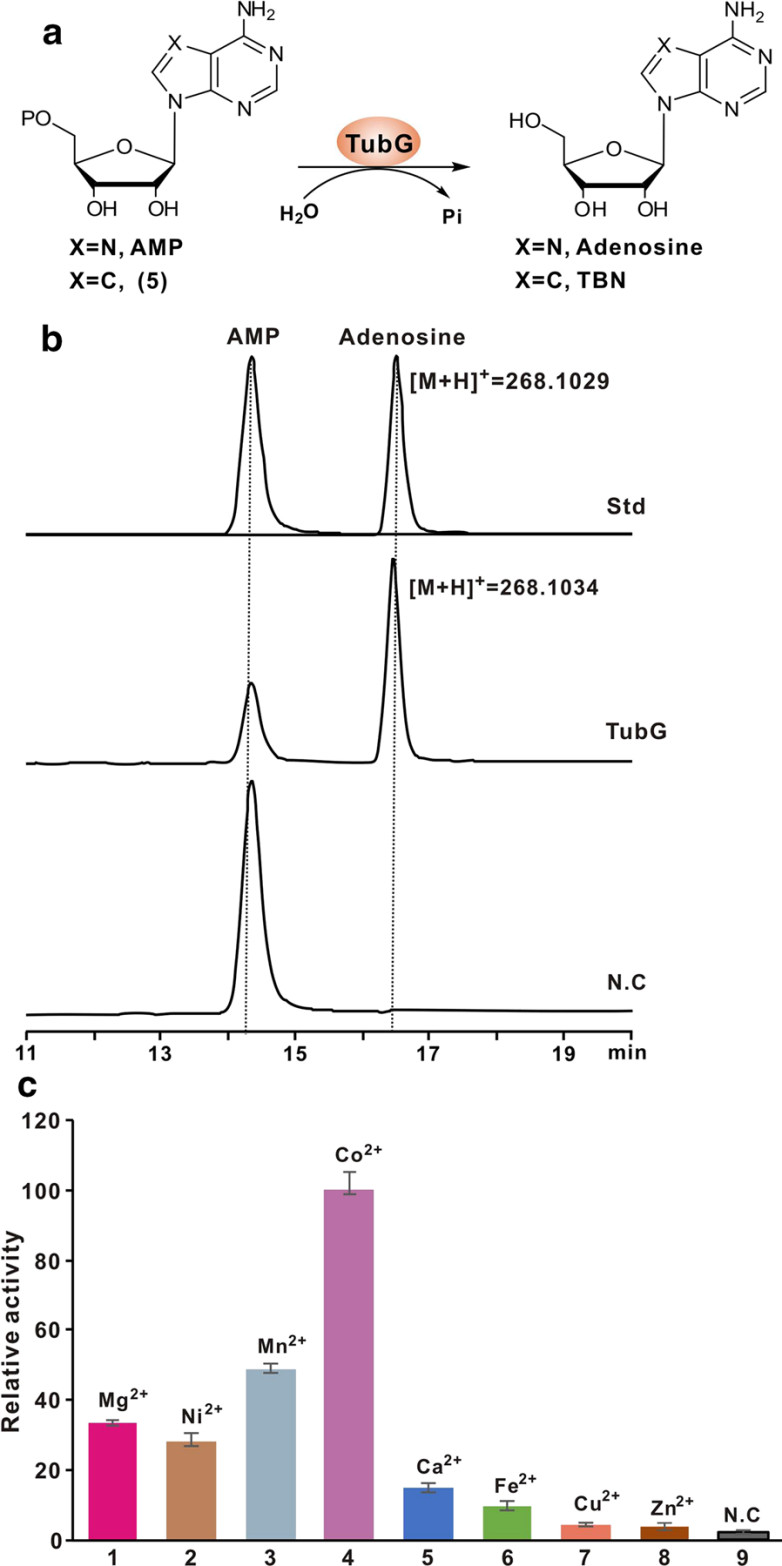
In vitro characterization of TubG as a Nudix hydrolase responsible for the final hydrolysis step. **a** Schematic of the TubG-catalyzed reaction using the analog of **5**, AMP, as substrate. **b** HPLC traces of the TubG reaction. Std, the authentic standards of AMP and adenosine; TubG, HPLC traces of the TubG reaction with AMP as substrate; N.C, EIC analysis of the reaction with AMP as substrate added but without TubG added. **c** Relative activity of TubG with AMP as substrate and different divalent cations as metallic cofactor

Bioinformatic analysis showed that TubG possesses a highly conserved 23-residue (GX5EX7REUXEEXGU) Nudix motif (Additional file 1: Figure S8) [21], which normally functions as a metal binding and catalytic site, accordingly, this protein is predicted to be a Nudix hydrolase for the final tailoring step during TBN biosynthesis. To determine if TubG executes the delineated functional role, it was overexpressed and purified from *E. coli* to near homogeneity (Additional file 1: Figure S9A). We then tested TubG activity using AMP (compound **5** analog) as an alternative substrate (Fig. 5a), and HPLC analysis indicated that the TubG catalyzed reaction produced the expected adenosine peak but not in the TubG-negative control (Fig. 5b). Further LC–MS results indicated that the peak could give rise to the target [M+H]^+^ ion at *m/z* 268.1034, whose fragmentation pattern is fully in accordance with that of the adenosine standard (Fig. 5b, Additional file 1: Figure S9B, C). In addition, we evaluated the divalent cations affecting enzymatic activity of TubG. Of all divalent cations (involving Mg^2+^, Ni^2+^, Mn^2+^, Co^2+^, Ca^2+^, Fe^2+^, Cu^2+^, and Zn^2+^) selected, we found that Co^2+^ is capable of maintaining the maximal enzymatic activity for TubG (Fig. 5c). All of this suggests that TubG is an atypical Nudix hydrolase using Co^2+^ as the most preferred divalent cation.

## Discussion

**Fig. 6:**
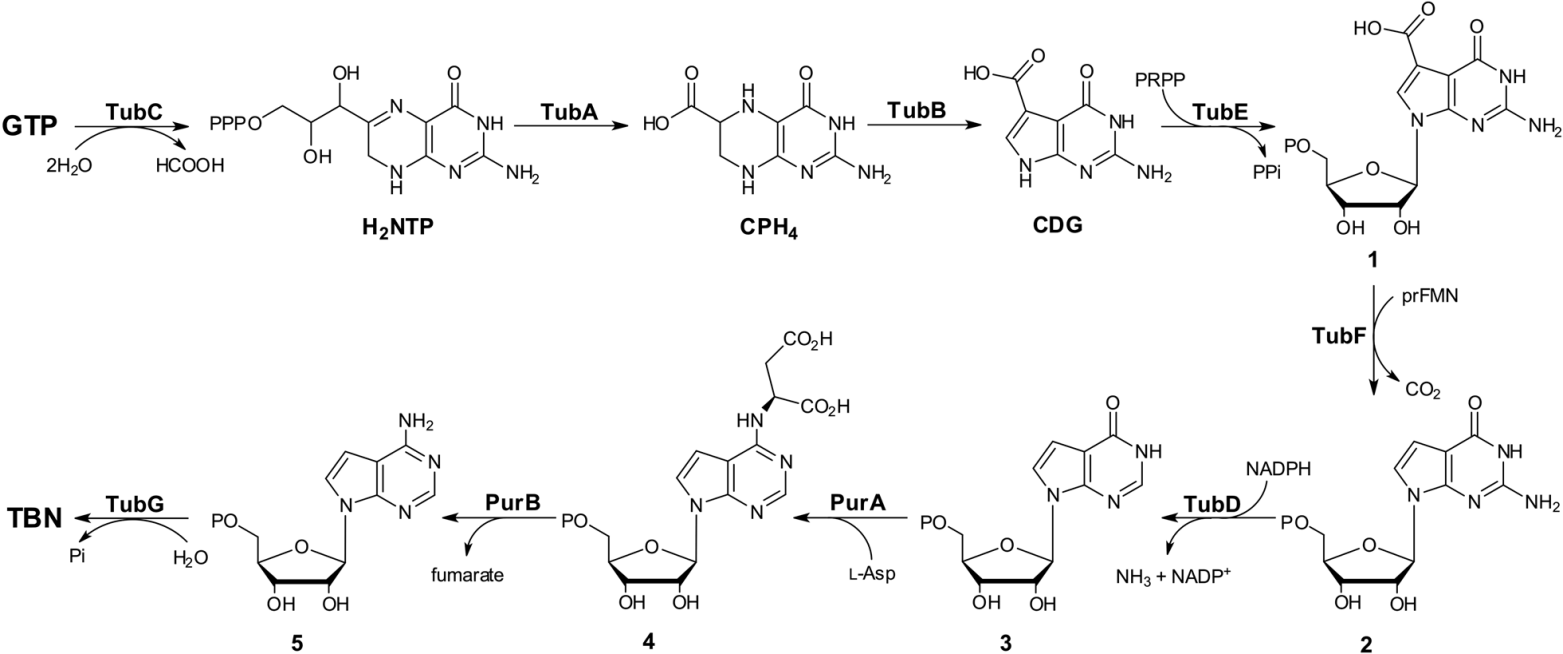
Proposed biosynthetic pathway to TBN. PurA, adenylosuccinate synthetase; PurB, adenylosuccinate lyase

Previous labeling and biosynthetic studies revealed that GTP is the direct substrate for the biosynthesis of the 7-deazapurine core in toyocamycin/sangivamycin, and a three-enzyme cascade, including GCH I (GTP cyclohydrolase I), CPH_4_ synthase (6-carboxyl-5,6,7,8-tetrahydropterin synthase), and CDG synthase (7-carboxy-7-deazaguanine synthase), accomplishes the biosynthesis of the 7-deazapurine core [10]. In the present study, this mechanism is also shown to be suitable for the building of TBN scaffold. Three enzymes, TubC (GCH I), TubA (CPH_4_ synthase), and TubB (CDG synthase), are found in the biosynthetic pathway to TBN. TubC is proposed to govern the initial step, catalyzing GTP transformation to H_2_NTP, which is subsequently converted to CPH_4_ by TubA (Fig. 6). TubB (CDG synthase) is proposed to employ a radical and Mg^2+^ dependent strategy to realize the radical-mediated ring contraction step in the course of CDG biosynthesis (Fig. 6).

Earlier bioinformatic analysis speculated that the 7-deazapurine core (7-cyano-7-deazaguanine/PreQ_0_) is biosynthesized preceding its assembly [2, 10]. In the present study, it seems to be logic and feasible in the TBN pathway. Two enzymes, TubE (PRPP transferase), and TubF (UbiD family decarboxylase), are demonstrated to be involved in the described enzymatic steps (Fig. 6). We initially deduced that CDG is likely to undergo decarboxylation prior to its condensation with PRPP for the accomplishment of assembly step, while TubE is not able to accept 7-deazaguanosine as the substrate. We therefore propose that CDG is first converted by TubE to form compound **1**, which is then decarboxylated to compound **2** by TubF, an unusual decarboxylase likely using prenylated-FMN (prFMN) as cofactor (Fig. 6).

For the tailoring steps during TBN biosynthesis, TubD (NADPH-dependent reductase) and TubG (Nudix hydrolase), which were characterized in the present study using natural substrate analogs, are established to participate in the enzymatic reactions, compound **2** (GMP analog) is converted to **3** (IMP analog). We could imagine that these two enzymes definitely show the preference to the natural substrates, however, it is also commonly acceptable to explore the catalytic mechanisms of certain enzymes with substrate analogs [22]. Indeed, enzymes are often rationally evolved to accept artificial substrate analogs for industrial biocatalysis purposes [22]. It is very interesting to ask why the enzymes for the sequential transformation of **3** to **4**, and to **5** are missing in the TBN pathway. Compound **3** is structurally similar to IMP; as a result, it is reasonable to propose that the missing two enzymes, adenylosuccinate synthetase (PurA), and adenylosuccinate lyase (PurB), could be certainly “borrowed” from the primary purine metabolic pathway (Fig. 6). As for compound **5,** it is confirmed to be hydrolyzed by TubG with removal of a phosphate for the accomplishment of TBN biosynthesis (Fig. 6).

There are two other enzymes, TubH (kinase), and TubI (MFS transporter), also present in the TBN pathway. It is more reasonable to propose that TubI is responsible for transporting TBN out of the cell once synthesized. With respect to TubH, the assignment of its functional role in TBN biosynthesis is highly challenging. Previous studies showed that TBN is toxic to *M. tuberculosis,* and accordingly, it is acceptable to hypothesize that this compound is also potentially toxic to the host cell. Microbes have developed several intricate strategies for the self-resistance of its secondary metabolites during the long-term evolution, undoubtedly, targeted modification (e.g. phosphorylation) of the target antibiotic should be a more effective and convenient tactic, and a similar case for antibiotic self-resistance by host cell has been previously reported in capuramycin biosynthesis [23]. From this perspective, we tentatively propose that TubH is likely to play a self-resistance role by phosphorylating TBN to relieve its potential toxicity to host cells, and related research is now underway.

Notably, the advent of rapid and affordable next-generation DNA sequencing has revolutionized and accelerated the traditional programs for the discovery of chemical diversities [24]. Therefore, TBN could be used as a promising template for the target-directed genome mining of related antibiotics, and we have already been rewarded with uncovering several potential TBN-related antibiotics pathways from the currently-available reservoir of the microbial genomes (Additional file 1: Figure S10). As a consequence, it would be of great interest in the future to hunt for novel 7-deazapurine-containing antibiotics by targeted genome mining approach.

## Conclusion

In summary, we report the discovery and functional analysis of the TBN biosynthetic pathway. We have determined that TBN biosynthesis employs a PRPP dependent strategy for the deazanucleoside scaffold assembly, and we have also provided the biochemical evidence that TubD and TubG are involved in the tailoring reduction and phosphohydrolysis steps. We anticipate that the deciphering of the biosynthetic puzzle of TBN will open the way for future combinatorial biosynthesis of this family of antibiotics for the rational generation of novel analogs with enhanced features.

## Methods

### Materials and general methods

Strains, plasmids used in this study are described in Additional file 1: Table S2, and PCR primers are listed in Additional file 1: Table S3. All of the enzymes (except for the DNA polymerase) were the products of New England Biolabs. The TBN standard was purchased from Medchem Express (MCE). Chemicals were purchased from Sigma-Aldrich, Thermo Scientific, J&K Scientific, or Sinopharm unless otherwise indicated. Standard protocols were employed to manipulate *E. coli* or *Streptomyces* according to those of Green et al. [25] or Kieser et al. [26].

### Sequencing analysis of the genome of *S. tubercidicus*

Genomic DNA of *S. tubercidicus* was isolated on the basis of the standard protocol [26], and the genome sequencing was performed using the Illumina Hiseq4000 sequencing system, and the assembled sequence data was then annotated using the Glimmer 3.02 software. The online programs FramePlot 4.0beta (http://nocardia.nih.go.jp/fp4/) and 2ndFind (http://biosyn.nih.go.jp/2ndfind/) were utilized for the accurate analysis of the TBN gene cluster.

### Accession numbers

The DNA sequence of the *tub* gene cluster is deposited in the GenBank database under accession number MG706975.

### Genomic library construction and screening for *S. tubercidicus*

For the construction of pJTU2463b-derived genomic library for *S. tubercidicus,* standard method was adopted, using the EPI300-T1R as suitable host cells, and a narrow-down PCR screening strategy [27] with primers TubidF/R were employed to screen the positive cosmid 12G4 from the genomic library.

### Fermentation of related *Streptomyces* strains for TBN production

For production of TBN, *Streptomyces* strains were inoculated in TSB medium and cultivated for 2 days, after that, the cultures (2%, V/V) were transferred to fermentation medium (including 20 g glucose, 30 g soluble starch, 10 g corn steep liquor, 10 g soybean meal, 5 g peptone, 2 g NaCl, 5 g CaCO_3_, pH 7.0) and fermented (180 r/min, 28 °C) for 5 days. Subsequently, the fermentation beer was processed (adding oxalic acid till pH 3.0) for LC–MS analysis.

### Overexpression and purification of TubE, TubD, and TubG in *E. coli* Rosetta (DE3)/pLysS

For overexpression of these proteins, their structural genes were amplified by KOD-plus DNA polymerase (TOYOBO) using related primers listed in Additional file 1: Table S3. Then the *Nde*I–*Eco*RI engineered DNA fragments were individually cloned into the counterpart sites of pET28a. After confirmation by sequencing, the related constructs were subsequently transformed into *E. coli* Rosetta (DE3)/pLysS cells according to the standard protocols [25]. Expression and purification for the His6-tagged proteins were conducted according to the method by Wu et al. [28].

### Enzymatic assays of TubE

For TubE activity, the reaction mixture (100 mM PBS buffer, pH 7.5; 1 mM CDG; 2 mM PRPP; 5 mM MgCl_2_; 1 mM DTT and 20 μg protein) was incubated at 30 °C for 8 h, and then terminated by the immediate addition of an equivalent volume methanol. After centrifugation to remove protein, the supernatant was filtrated by 0.22 μm filter. HPLC (Shimadzu LC-20A) analysis was performed on a reverse phase C18 column (Inertsil ODS-3, 4.6 × 250 mm, 5 µm) with the elution gradient of 5%–30% methanol:0.15% TFA over 35 min at a flow rate of 0.5 ml/min, and the elution was monitored at UV295 nm by a DAD detector.

### Enzymatic assays of TubD

For TubD activity, the reaction containing 100 mM Tris–HCl buffer (pH 7.5), 1 mM GMP, 1 mM NADPH/NADH, 1 mM DTT, 50 mM KCl, 2 mM EDTA, and 20 μg protein, incubated at 30 °C for 4 h, then the supernatant was analyzed by LC–HRMS with the elution gradient of 5%–25% methanol:0.15% TFA over 20 min at a flow rate of 0.4 ml/min.

### Enzymatic assays of TubG

The complete TubG reaction including 100 mM Tris–HCl buffer (pH 7.0), 1 mM AMP, 100 mM KCl, 5 mM divalent ion (Mg^2+^, Ni^2+^, Mn^2+^, Co^2+^, Ca^2+^, Fe^2+^, Cu^2+^, and Zn^2+^), and 20 μg protein, was incubated at 30 °C for 4 h. The protein in reaction was removed by adding an equal volume of methanol, then the supernatant was analyzed by HPLC (Shimadzu LC-20A) equipped with C-18 reversed-phase column (Inertsil ODS-3, 4.6 × 250 mm, 5 µm) with 5%–30% methanol:0.15% TFA over 15 min at a flow rate of 0.4 ml/min, the condition (30% methanol:0.15% TFA) was maintained for another 10 min.

### The conditions for LC–MS analysis

LC–MS analysis was carried out on a Thermo Fisher Scientific ESI-LTQ Orbitrap (Scientific Inc.) equipped with a C-18 reversed-phase column (Inertsil ODS-3, 4.6 × 250 mm, 5 µm) in positive mode with an elution gradient of 5%–30% Methanol:0.15% TFA over 30 min at 0.5 ml/min, and the parameters for the LC–MS analysis are as follows: Dry gas at 275 °C, 10 l/ml, and nebulizer pressure of 30 psi.

## Additional file

**Additional file 1: Figure S1.** LC-MS analysis of the target metabolite produced by *S. coelicolor* M1154/12G4. **Figure S2.** Bioinformatic analysis of the phosphoribosyltransferase TubE with its homologs. **Figure S3.** Biochemical characterization of TubE reaction. **Figure S4.** ^1^H and ^13^C NMR analysis of the compound **1**. **Figure S5.** ^1^H-^1^H cosy, HMBC, and HSQC spectrums of the compound **1. Figure S6.** Bioinformatic analysis of the GMP reductase TubD with its homologs. **Figure S7.** Biochemical characterization of TubD-catalyzed reaction. **Figure S8.** Bioinformatics analysis of the Nudix superfamily hydrolase TubG. **Figure S9.** Biochemical analysis of TubG and its reaction. **Figure S10.** Target-directed genome mining of the gene clusters for the potential TBN-related antibiotics. **Table S1.** Parameters for the genome sequencing and assembly of *S. tubercidicus*. **Table S2.** Strains, plasmids and cosmids used in this study. **Table S3.** PCR primers used in this study.
