# Supplementary material for: Discovery and characterization of the tubercidin biosynthetic pathway from Streptomyces tubercidicus NBRC 13090
Source: Microb Cell Fact. 2018 Aug 28;17:131. doi: 10.1186/s12934-018-0978-8 (PMC6112128; doi:10.1186/s12934-018-0978-8)
Supplement: Supplementary file 1 — Additional file 1: Figure S1. LC-MS analysis of the target metabolite produced by S. coelicolor M1154/12G4. Figure S2. Bioinformatic analysis of the phosphoribosyltransferase TubE with its homologs. Figure S3. Biochemical characterization of TubE reaction. Figure S4. 1H and 13C NMR analysis of the compound 1. Figure S5. 1H-1H cosy, HMBC, and HSQC spectrums of the compound 1. Figure S6. Bioinformatic analysis of the GMP reductase TubD with its homologs. Figure S7. Biochemical characterization of TubD-catalyzed reaction. Figure S8. Bioinformatics analysis of the Nudix superfamily hydrolase TubG. Figure S9. Biochemical analysis of TubG and its reaction. Figure S10. Target-directed genome mining of the gene clusters for the potential TBN-related antibiotics. Table S1. Parameters for the genome sequencing and assembly of S. tubercidicus. Table S2. Strains, plasmids and cosmids used in this study. Table S3. PCR primers used in this study. [file 12934_2018_978_MOESM1_ESM.pdf]

Discovery and characterization of the tubercidin biosynthetic pathway from *Streptomyces tubercidicus* NBRC 13090

Yan Liu[1], Rong Gong[2], Xiaoqin Liu[2], Peichao Zhang[2], Qi Zhang[1], You-Sheng Cai[2], Zixin Deng[2], Margit Winkler[3], Jianguo Wu[1,*], Wenqing Chen[2,*]

[1]State Key Laboratory of Virology, and College of Life Sciences, Wuhan University, Wuhan 430072, China.

[2]Key Laboratory of Combinatorial Biosynthesis and Drug Discovery, Ministry of Education, and School of Pharmaceutical Sciences, Wuhan University, Wuhan 430071, China.

[3]Austrian Centre of Industrial Biotechnology, Petersgasse 14, 8010 Graz, Austria.

[*]For Correspondence: **Wenqing Chen**, School of Pharmaceutical Sciences, Wuhan University, Wuhan 430071, China. **E-mail**: wqchen@whu.edu.cn, or **Jianguo Wu**, State Key Laboratory of Virology, and College of Life Sciences, Wuhan University, Wuhan 430072, China. **E-mail**: jwu@whu.edu.cn

Running Title: the tubercidin biosynthetic pathway

1. Additional Figures

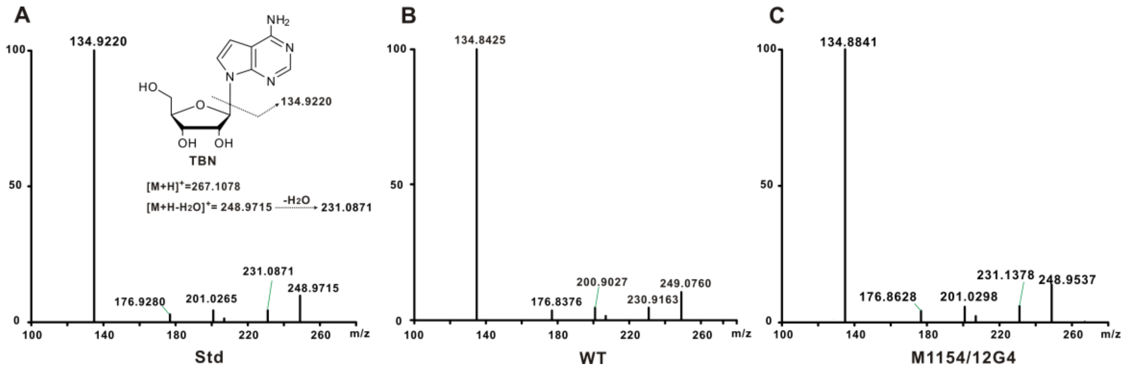

Figure S1. LC-MS analysis of the target metabolite produced by *S. coelicolor* M1154/12G4.

(A) MS/MS analysis of the TBN authentic standard. The fragmentation pattern of TBN is also shown in this panel. (B) MS/MS analysis of the target metabolite produced by the recombinant *S. coelicolor* M1154/12G4. (C) MS/MS analysis of TBN produced by *S. tubercidicus* NBRC 13090.

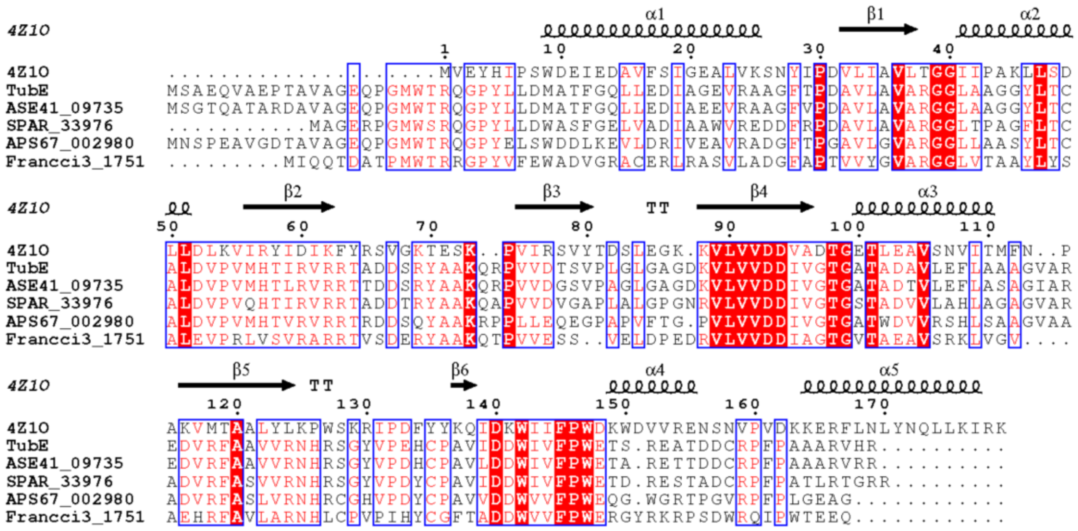

Figure S2. Bioinformatic analysis of the phosphoribosyltransferase TubE with its homologs.

Proteins include TubE (*S. tubercidicus* NBRC 13090), 4Z1O (*Sulfolobus solfataricus*, PDB: 4Z1O_A), ASE41_09735 (*Streptomyces* sp. Root264, Accession no: KRD23283.1), SPAR_33976 (*S. sparsogenes* DSM 40356, Accession no: OMI34940.1), APS67_002980 (*Streptomyces* sp. AVP053U2, Accession no: ODA72681.1), Francci3_1751 (*Frankia casuarinae*, Accession no: ABD11127.1). The sequences were aligned by ClustalW and merged with ESPript online program.

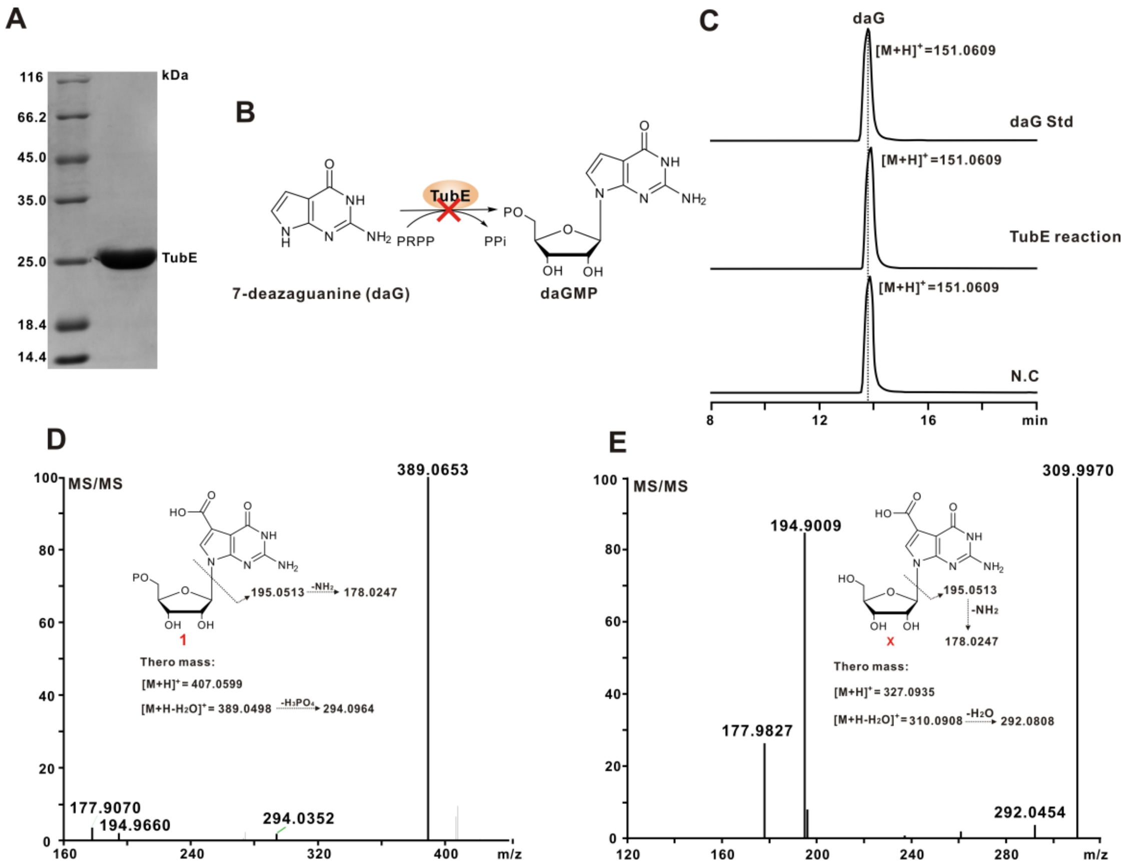

Figure S3. Biochemical characterization of the TubE reaction.

(A) SDS-PAGE analysis of the purified TubE from *E. coli*. (B) Schematic of the TubE-catalyzed reaction with daG as potential substrate. (C) Extracted ion chromatogram (EIC) analysis of the substrate daG and the deduced product daGMP. As indicated in Figure S3C, the target $[M+H]^+$ ion of daG could only be detected from the TubE reaction, suggesting that this compound is not the substrate of TubE. (D) MS/MS analysis of the compound **1**. The fragmentation pattern is also shown in this panel. (E) MS/MS analysis of the target metabolite of the compound **X**. As indicated, the main fragment ions of **X** are well-matched to the theoretical pattern of dephosphorylated form of **1**, suggesting that **X** is spontaneously produced in the process of HPLC analysis.

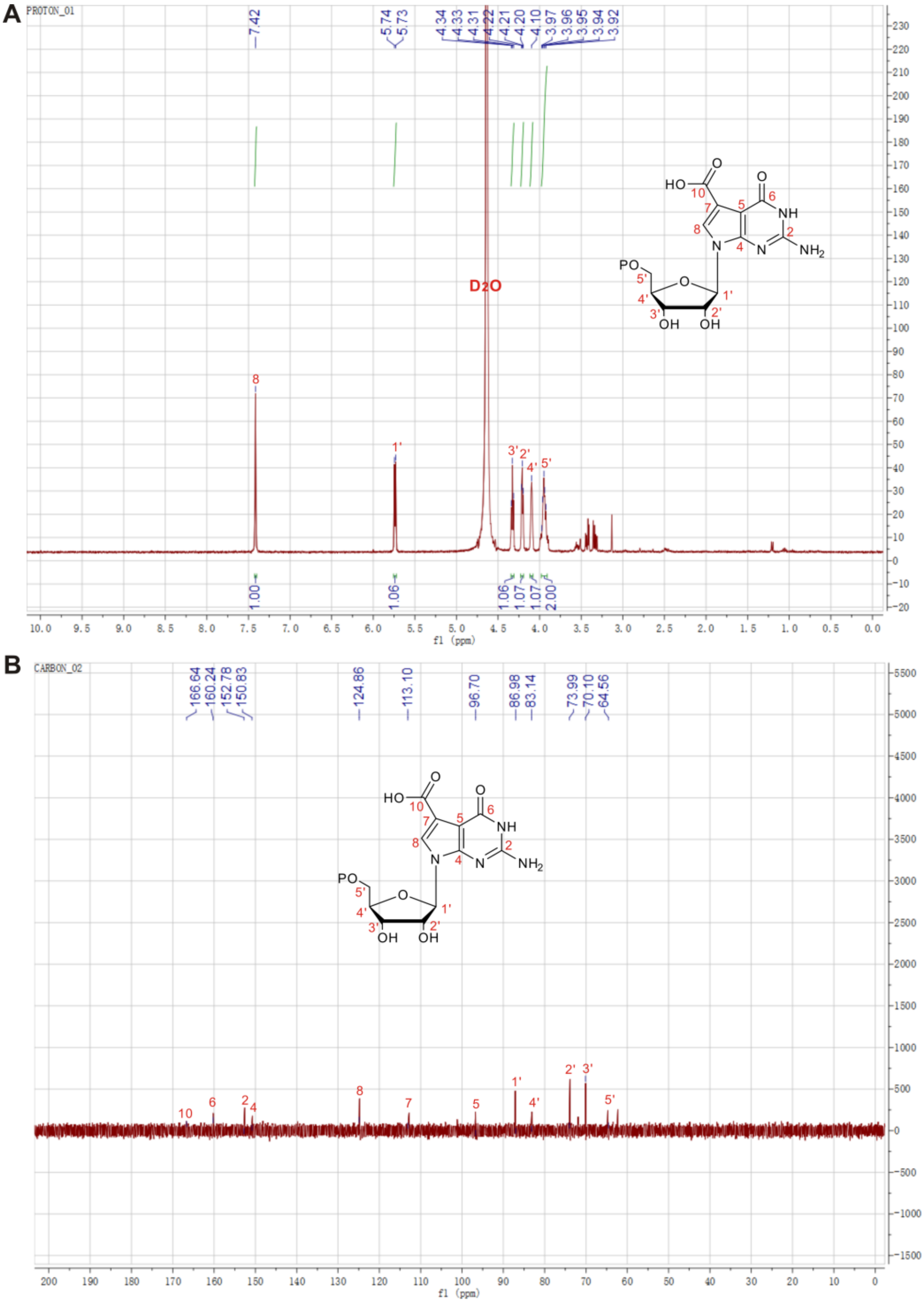

Figure S4. ^{1}H and ^{13}C NMR analysis of the compound 1.

(A) ^{1}H-NMR spectrum of compound **1** (600 MHz, D_2O). The ^{1}H NMR data of compound 1 is listed as follows: ^{1}H NMR (600 MHz, D_2O) δ 7.42 (1H, s), 5.74 (1H, d, *J* =6.0 Hz), 4.33 (1H, t, *J*=18.0 Hz), 4.22 (1H, t, *J*=12.0 Hz), 4.10 (1H, m), 3.97-3.92(2H,m). (B) ^{13}C NMR spectrum of compound **1**. ^{13}C NMR data of compound **1** is indicated as follows: ^{13}C NMR (600 MHz, D_2O) δ 166.64, 160.24, 152.78, 150.83, 124.86, 113.10, 96.70, 86.98, 83.14, 73.99, 70.10, 64.56.

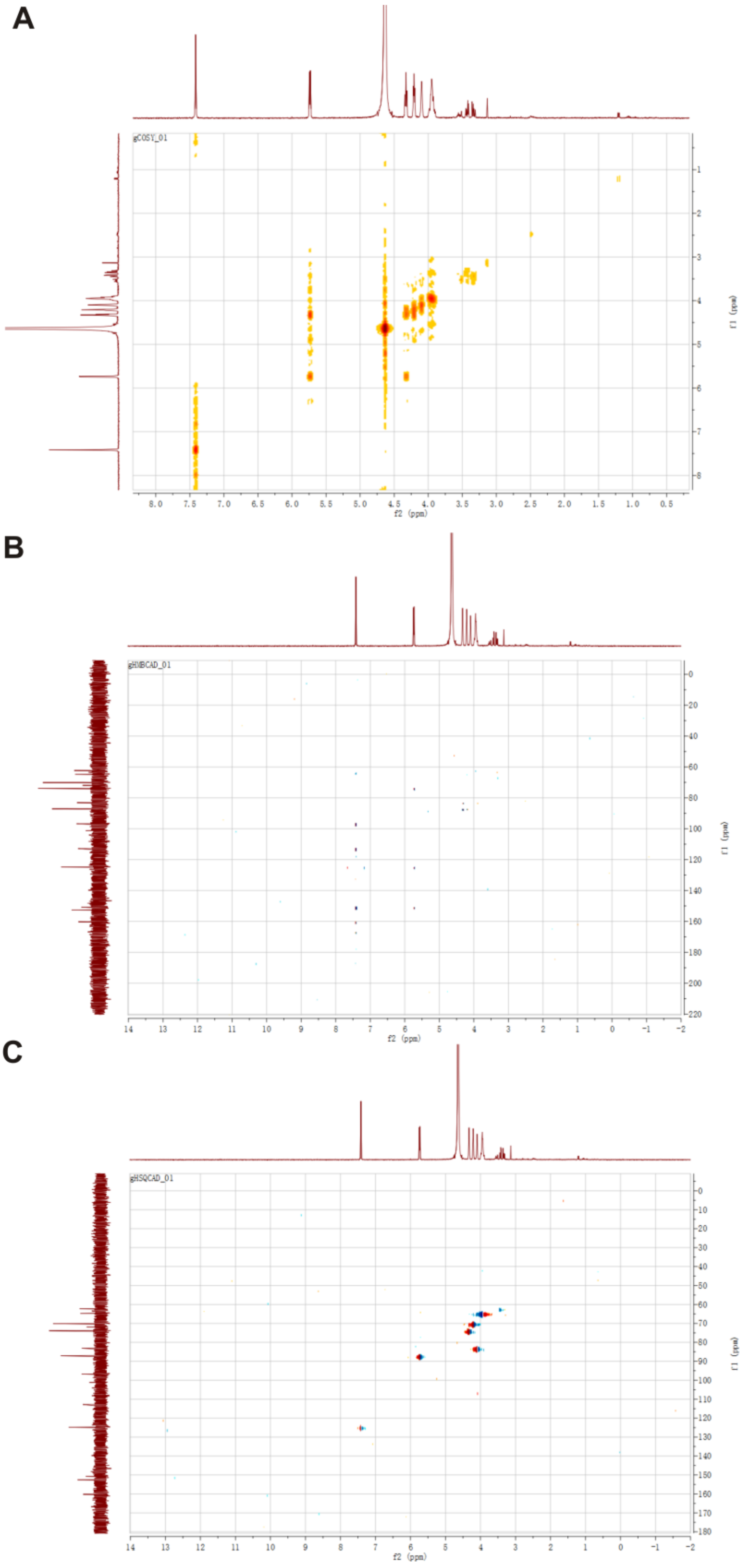

Figure S5. ^{1}H-^{1}H cosy, HMBC, and HSQC spectrums of the compound 1.

(A) ^{1}H-^{1}H cosy spectrum of compound **1** (600 MHz, D_2O). (B) HMBC spectrum of compound **1** (600 MHz, D_2O). (C) HSQC spectrum of compound **1** (600 MHz, D_2O).

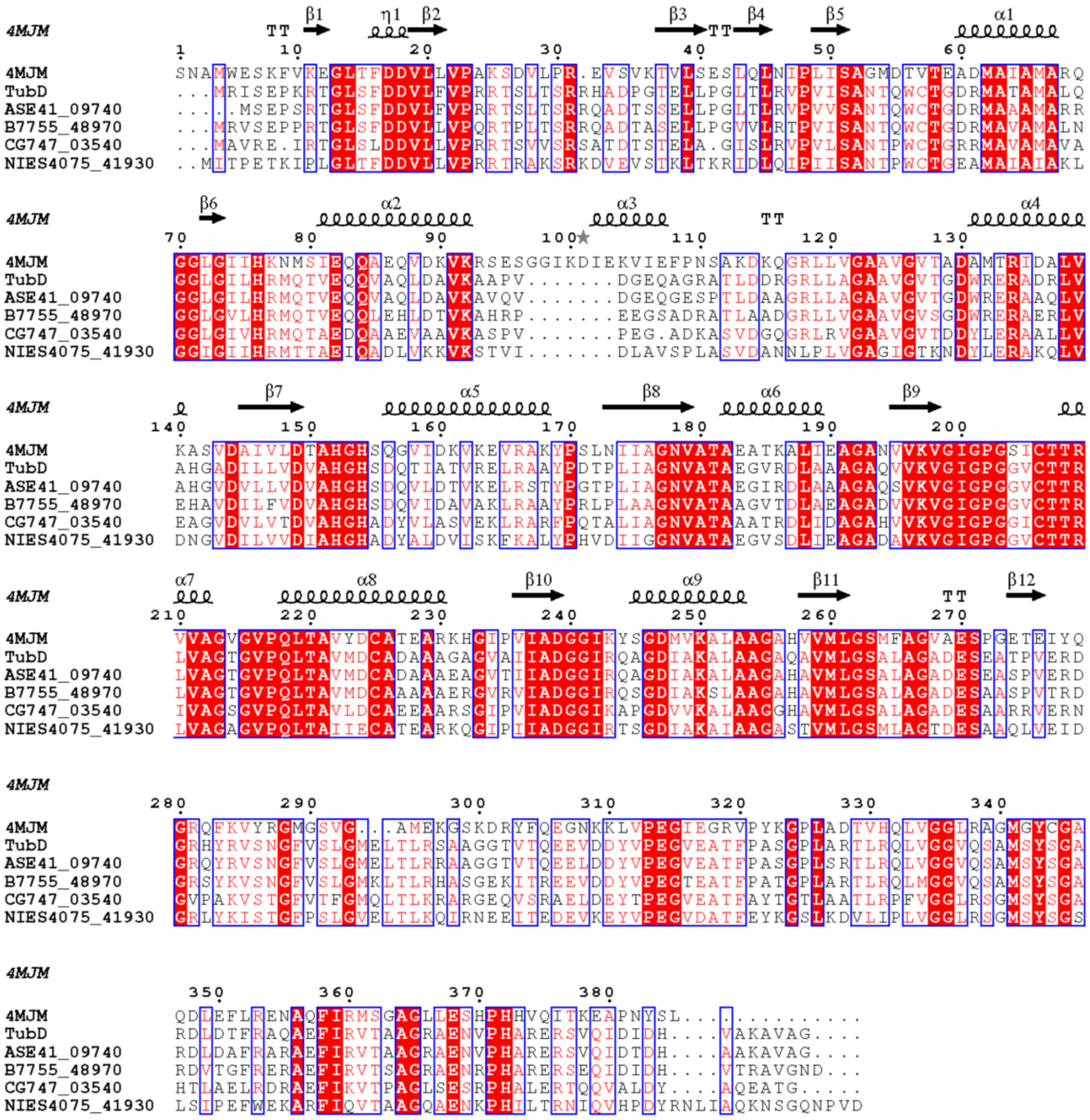

Figure S6. Bioinformatic analysis of the GMP reductase TubD with its homologs.

The detailed information for TubD and its homologs is as follows: 4MJM is from *Bacillus anthracis* Str. Ames (PDB: 4MJM_A), ASE41_09740 is from *Streptomyces* sp. Root264 (Accession no: KRD23537.1), B7755_48970 is from *Streptomyces* sp. NBS 14/10 (Accession no: OXL22932.1), CG747_03540 is from *Streptomyces* sp. CB02959 (GenBank: PJN41753.1), and NIES4075_41930 is from *Tolypothrix* sp. NIES-4075. The sequences were aligned by ClustalW and merged with ESPript online program.

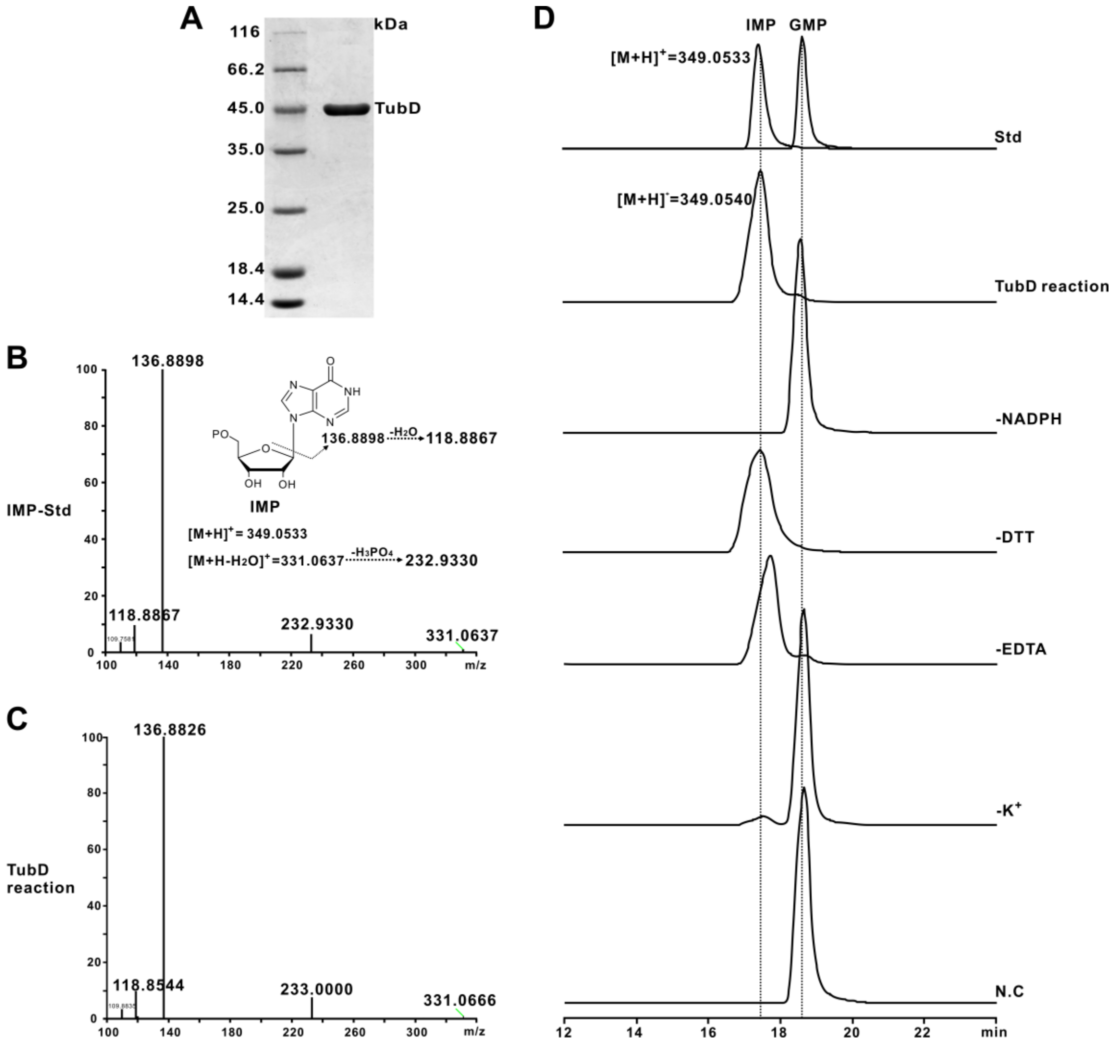

Figure S7. Biochemical characterization of TubD-catalyzed reaction.

(A) SDS-PAGE analysis of the purified TubD from *E. coli*. (B) MS/MS analysis of the IMP (inosine monophosphate) authentic standard. The fragmentation pattern is also indicated in this panel. (C) MS/MS analysis of the target metabolite of TubD-catalyzed reaction. (D) Biochemical determination of the factors that potentially affects the enzymatic activities of TubD. TubD reaction, the complete TubD reaction; -NADPH, the TubD reaction without NADPH added; -DTT, the TubD reaction without DTT added; $-K^+$, the TubD reaction without K^+ added; N.C, the reaction without TubD added as negative control.

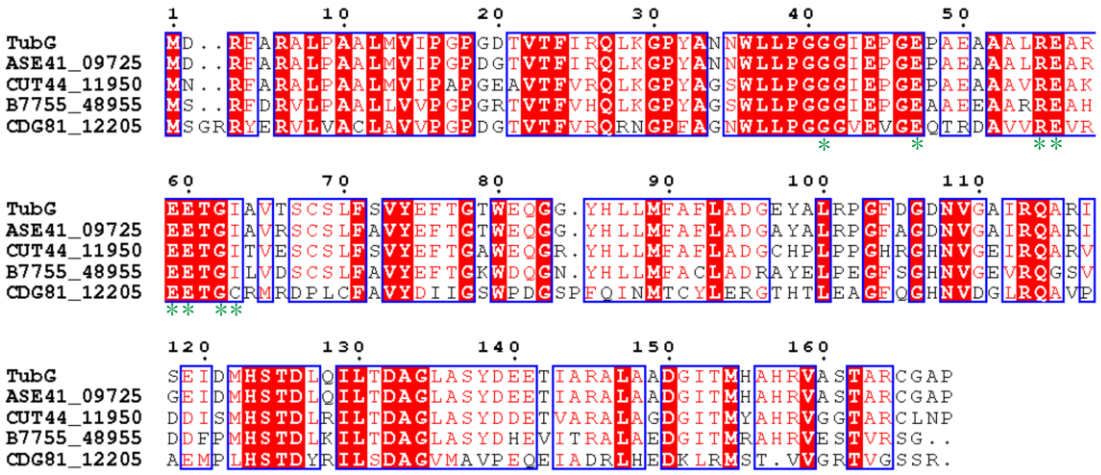

Figure S8. Bioinformatics analysis of the Nudix superfamily hydrolase TubG.

Bioinformatics analysis of TubG with its homologs. Proteins include TubG (*S. tubercidicus* NBRC 13090), ASE41_09725 (*Streptomyces* sp. Root264, Accession no: KRD23281), CUT44_11950 (*Streptomyces* sp. TRM SA0054, Accession no: PJE97395), B7755_48955 (*Streptomyces* sp. NBS 14/10, OXL22929), and CDG81_12205 (*Actinopolyspora erythraea*, ASU78919). These sequences are aligned by ClustalW and merged with ESPript online program. The conserved NUDIX motif “GX_5EX_7REUXEEXGV” was highlighted with green asterisks (*) at the bottom of the conserved residues.

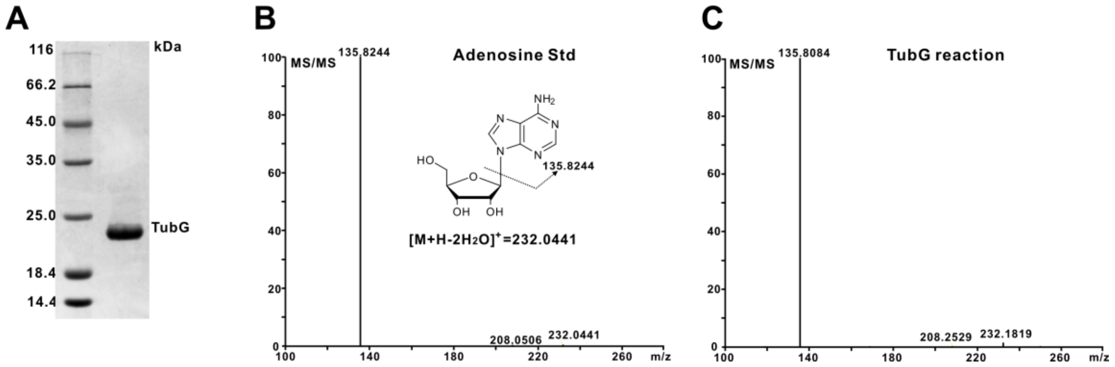

Figure S9. Biochemical analysis of TubG and its reaction.

(A) SDS-PAGE analysis of the purified TubG overexpressed in *E. coli*. (B) MS/MS analysis and fragmentation pattern of the adenosine authentic standard. (C) MS/MS analysis of the target product from TubG reaction using AMP as substrate.

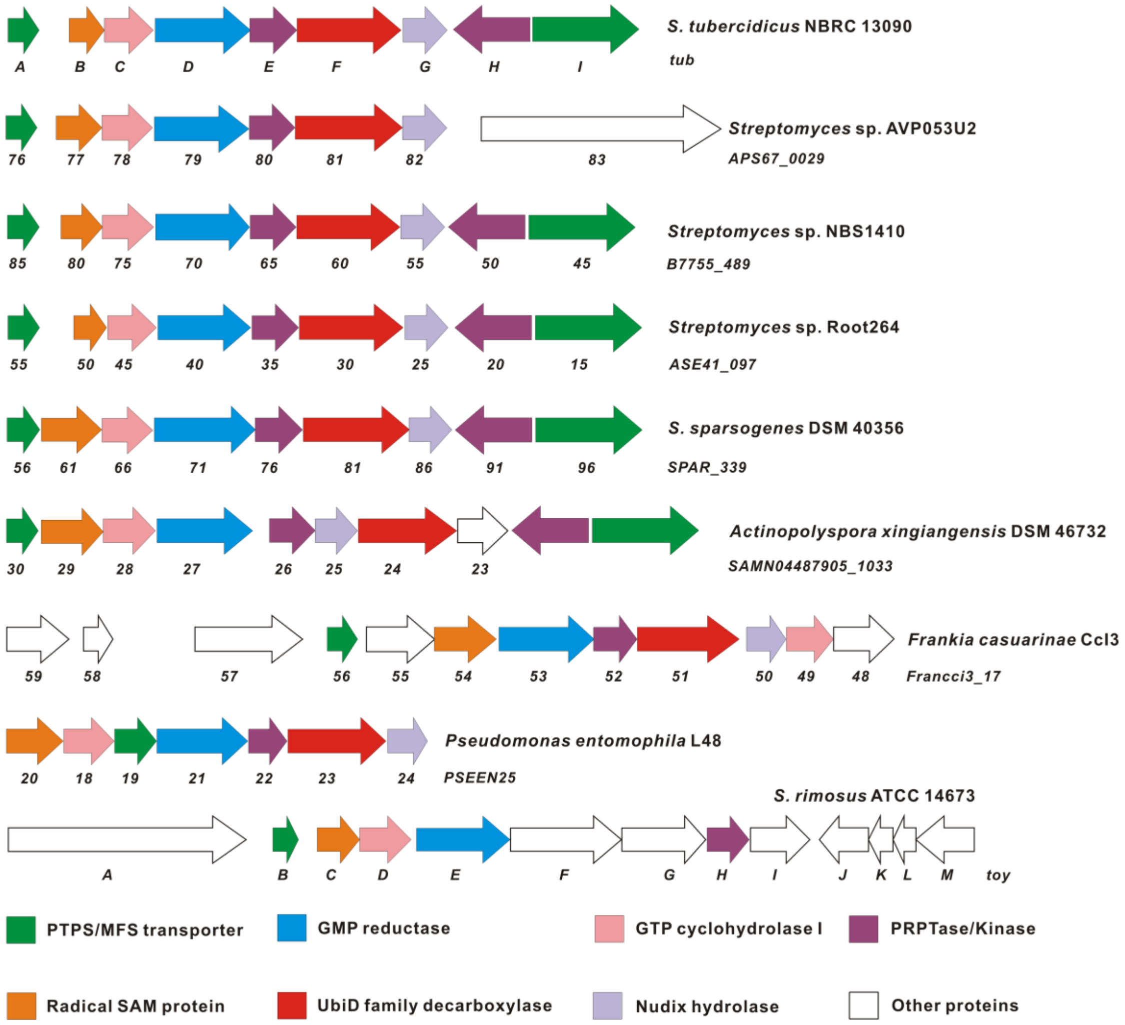

Figure S10. Target-directed genome mining of the gene clusters for the potential TBN-related antibiotics.

The potential gene clusters of the TBN-related antibiotics were obtained using the TubA, TubB, and TubC as probes. As indicated here, the proposed functions for the conserved proteins are highlighted with corresponding colors.

2. Additional Tables

Table S1. Parameters for the genome sequencing and assembly of *S. tubercidicus*.

**Sample**	***S. tubercidicus* NBRC 13090**
No. of all scaffolds	38
Bases in all scaffolds	7888449
No. of large scaffords(> 1000 bp)	22
Bases in large scaffolds	7880373
Largest length	1607930
Scaffold N50	1309713
Scaffold N90	358127
G+C content	70.78
N rate	0.00679
No. of all contigs	53
Bases in all contigs	7887913
No. of large contigs(> 1000 bp)	36
Bases in large contigs	7878900
Largest length	1302292
Contig N50	783438
Contig N90	298401
Gene num	7263

Table S2. Strains, plasmids and cosmids used in this study.

Strain/ Plasmid / Cosmid	Relevant characteristics	Reference or source
**Strain**		
S. tubercidicus		
NBRC 13090	Wild-type producer for tubercidin	[1]
S. coelicolor M1154	*S. coelicolor* A3(2) derivative used as heterologous host	[2]
M1154/12G4	*S. coelicolor* M1154 derivative containing cosmid 12G4	This study
M1154/pJTU2463b	*S. coelicolor* M1154 derivative containing pJTU2463b	This study
E. coli		
DH10B	F^-, *mcrA*, Δ(*mrr-hsd*RMS-*mcr*BC), ϕ80d, *lac*ZΔM15, Δ*lac*X74, *deo*R, *rec*A, *lend*A, *lara*Δ139, D(*ara*, *leu*)7697, *gal*U, *gal*K, λ^-, *rps*L, *nup*G	Gibco-BRL
Rosetta(DE3)/pLysS	F^-, *ompT*, $hsdS_B(r_B^- m_B^-)$, gal, *dcm* λ(DE3 [lacI lacUV5-T7 gene1 ind1 sam7 nin5]) pLysS (CmI^R)	Novagen
ET12567(pUZ8002)	F^-, dam^-, 13::Tn9, dcm^-6, *hsd*M, *hsd*R, recF143, zjj-202::Tn10, galK2, galT22,ara14, pacY1, xyl-5,leuB6, thi-1, pUZ8002	[3]
EPI300-$T1^R$	host cell for genomic library construction	Epicenter
**Plasmids**		
pEASY-Blunt	pUCori, *lacZ*, f1 *ori*, *neo*, *bla*	TransGen Biotech
pJTU2463b	*int*, *aac(3)IV*, *ori*T, RK2, phiC31, *attP*	[4]
pET28a	*neo*, rep^{pMB1}, T7 promoter	Novagen
pET28a/*tubE*	pET28a derivative with an engineered NdeI-EcoRI fragment containing *tubE* cloned into counterpart sites	This study
pET28a/*tubD*	pET28a derivative with an engineered NdeI-EcoRI fragment containing *tubD* cloned into counterpart sites	This study
pET28a/*tubG*	pET28a derivative with an engineered NdeI-EcoRI fragment containing *tubG* cloned into counterpart sites	This study
12G4	pJTU2463b derived cosmid containing the entire TBN biosynthetic gene cluster	This study

Table S3. PCR primers used in this study.

Primers	Sequence (5'--3')
TubidF	CGAGGGCGACGACTACGAGA
TubidR	CGGGTGGTGAAGCCATAAGT
2463seqF	GCCATAGAGGGGCGTCGTG
2463seqR	TAAGTGCGGCGACGATAGT
TubDexF	gtccat ATGCGAATCTCCGAACCGA
TubDexR	ggaatTCATCCCGCCACCGCCTT
TubEexF	gtccat ATGAGCGCGGAACAGGTCG
TubEexR	ggaatTCACCGGTGCACCCGCGCCG
TubGexF	gtccat ATGGACCGATTCGCGCGAG
TubGexR	ggaatTCACGGCGCGCCGCACCGG

4. Additional References

1. Smulson ME, Suhadolnik RJ: **The biosynthesis of the 7-deazaadenine ribonucleoside, tubercidin, by *Streptomyces tubercidicus*.** *J Biol Chem* 1967, **242:**2872-2876.
2. Gomez-Escribano JP, Bibb MJ: **Engineering *Streptomyces coelicolor* for heterologous expression of secondary metabolite gene clusters.** *Microb Biotechnol* 2011, **4:**207-215.
3. Kieser T, Bibb MJ, Chater KF, Butter MJ, Hopwood. DA: *Practical Streptomyces Genetics* 2000:2nd ed., John Innes Foundation, Norwich, United Kingdom.
4. Cheng L, Chen W, Zhai L, Xu D, Huang T, Lin S, Zhou X, Deng Z: **Identification of the gene cluster involved in muraymycin biosynthesis from *Streptomyces* sp. NRRL 30471.** *Mol Biosyst* 2011, **7:**920-927.
